# Management of the 2012 *Legionella* crisis in Quebec City: need for a better communication between resources and knowledge transfer

**DOI:** 10.3389/fmicb.2014.00182

**Published:** 2014-05-05

**Authors:** Luc Trudel, Marc Veillette, Laetitia Bonifait, Caroline Duchaine

**Affiliations:** ^1^Département de Biochimie, Microbiologie et Bio-Informatique, Faculté des Sciences et de Génie, Université LavalQuébec, QC, Canada; ^2^Research in Pulmonary Medicine, Centre de Recherche De l'Institut Universitaire de Cardiologie et de Pneumologie de QuébecQuébec, QC, Canada

**Keywords:** *Legionella pneumophila*, legionellosis, cooling towers, outbreak, water sampling

## Introduction

*Legionella pneumophila* is one of the few bacteria that can be considered as a genuine environmental pathogen. Whilst most infections of hydric origins result from the faecal pollution of a stream or ground water, it is indeed not the case for *Legionella pneumophila* since this bacterium can be found in an ubiquitous manner in fresh water where it can survive temperature variations from 5.7 to 63°C (Fliermans et al., 1981). Consequently, any machinery or device using a water supply can be colonized with *Legionellae*, especially if the water temperature is high as it favor its growth: cooling towers, plumbing, water-heaters and hot tubs are few examples.

Between July 18th and October 8th, 2012, 181 cases of legionellosis have been reported in the Quebec City area, 14 of which being sadly fatal. The investigation done by the Direction de la santé publique (DSP) (public health management office) assisted by the Ministère du Développement durable, de l'Environnement, de la Faune et des Parcs (MDDEFP) (Ministry of sustainable development, environment, fauna and parks) and the Institut de recherche Robert Sauvé en santé et sécurité du travail du Québec (IRSST) (Robert Sauvé occupational health and safety research institute) has been long and tedious for various reasons that will be discussed later on. This extended delay between first case notification and resolution of crisis has enticed the media to spread messages, sometimes contradictory, and to give the floor to pseudo-experts who, by proposing, for example, source of outbreak that were totally improbable in these circumstances, needlessly alarmed and increase the panic response of the population (Pelchat et al., 2012).

It was people living in or frequently visiting the St-Roch and Limoilou districts (lower part of the city) that were contaminated by this strain of *Legionella* and, from the first notified cases, the cooling towers found in the area were suspected of harboring the pathogenic strain (Isabelle Goupil Sormany, 2012). These towers are essentially heat exchangers between the water and ambient air. The water to be cooled, the temperature of which usually varies between 25 and 40°C, is pulverized upward in the cooling tower using forced ventilation, loading the air released by the tower with steam created by the evaporation stream and tiny droplets which are the preferred conveyers for this pulmonary pathogen (Keller et al., 1996). The factors known to favor the proliferation of legionellae are the temperature (25–40°C), stagnancy, presence of sediments, scale, biofilms and corrosion, as well as the presence of amoebas and ciliate protozoans that could support the *Legionella* intracellular growth, all conditions found in cooling towers during summertime (Buse et al., 2012).

## History of an outbreak

**Figure 1:**
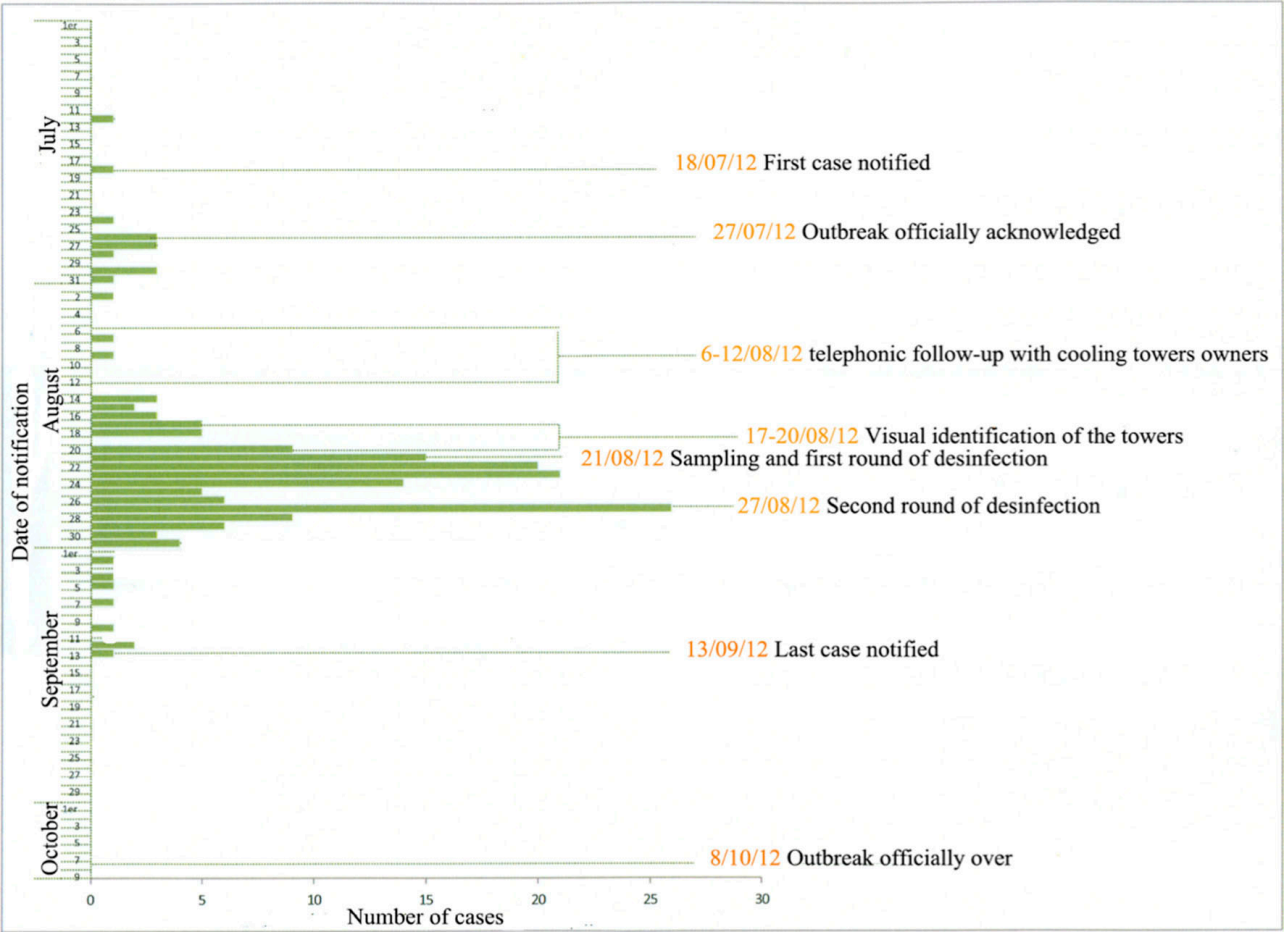
**Important dates and actions during the 2012 legionellosis outbreak in Quebec (modified from the “Rapport du directeur de la santé publique, François Desbiens, M.D. Éclosion de légionelles dans la ville de Québec”)**.

The report from the DSP published after the Quebec City 2012 legionellosis outbreak (Isabelle Goupil Sormany, 2012) is a precious information source when trying to explain the extended delay between the first cases and the resolution of the crisis. Figure 1 shows the evolution of the situation and lists key dates of the outbreak of *Legionella* in Quebec City during summer 2012.

From this report, we can first learn the regulated procedures in case of such outbreaks. Indeed, the Public Health Law allows the DSP to proceed with an epidemiologic investigation in any situation where there is serious motives to believe the public health is or could be threaten.

Therefore the DSP set in motion an epidemiologic investigation.

Whilst an intervention guide on *Legionella* (Décarie et al., 2010) mentioning that the “control intervention on the source should be done as soon as possible” was published in 2010 by the health authorities, it does not provide the following precisions:

1. How the validation of the cooling towers maintenance should be done;
2. At what distance from the location of the clinical case should the samples be taken when no source has been clearly identified;
3. How the water sampling in the cooling towers should be performed;
4. Where should the samples be forwarded (although it is clearly suggested to proceed with a service agreement with the Quebec Public Health Laboratory (LSPQ);
5. How the results should be interpreted.

Furthermore, this guide provides no detail regarding the treatment that could help control the *Legionella* contamination in a cooling tower during such situation. It is therefore understandable that the people who had to intervene were resource-less due to the lack of information.

Hence, from August 2nd, the DSP proceeded with a first wave of intervention entitled « voluntary measures » during which the building's owners were made aware of the problem, through both a media campaign and individual mail contact, and encouraged to proceed to a water quality control and thorough cleaning of their installations. A second wave of intervention, entitled « mandatory measures » was set off as of August 14th and aimed at:

1. Identify the cooling towers in the area where the highest number of affected people were found;
2. Identify the contamination source using water samples;
3. Perform a visual evaluation of the cooling towers maintenance;
4. Proceed to the sanitization whilst awaiting the analyses results;
5. Prescribe control measures according to the results obtained from the water samples and observations from the cooling towers inspections.

The sampling and sanitization treatments of the cooling towers were initiated on August 21st. It therefore took over a month before proceeding to the first inspections aimed at identifying and sanitizing the source or sources responsible for this outbreak. Why such a delay? Several assumptions can be made:

- Any governmental machine is weighty and complicated to get started;
- The authorities were not ready to face this crisis;
- There were no inventory, nor maintenance registry of the cooling towers even if so recommended by several reports dating from the previous Quebec City *Legionella* outbreak in 1997;
- Legally, it is impossible to proceed with any sampling in private buildings without being mandated to do so.

The analysis of the samples was done by the MDDEFP and the IRSST and both used culture to evaluate the concentration of *Legionella* found in various cooling towers. This approach, according to the Public Health Director, requires 20 days before any information on the quantity and the exact identity of the *Legionella* strains can be obtained. These 20 days added to the 30 or so spent prior to the beginning of the analyses and you end up with more than 50 days from the start of the outbreak to the first experimental results pointing toward a potential source. Even though a first round of disinfection of the towers has been initiated on August 21st, a survey made 1 week later showed that 21% of the towers still shelter significant concentrations of *Legionella*.

## Could it have been done better… or differently?

The answer is definitely YES. The delay of almost 2 months could have been noticeably reduced if academic or private research laboratories had been involved from the start. What would have been the advantages of consulting and using the expertise found locally or internationally?

Several research laboratories own the necessary tools to perform rapid molecular analyses for the quantification and identification of legionellae as well as characterization of water microbial flora. These research methods, whilst non-standardized or validated as those routinely used by the Quebec government laboratories or any other reference laboratory, are extremely rapid, accurate and powerful and would have allowed for an answer regarding either the presence of *Legionella* or their concentration. Several reports state that real time PCR and its derivative, viable qPCR, have immense potential for the accurate, rapid and cost-effective detection and enumeration of *Legionella* in environmental samples and can be used as a complementary tool for the detection and monitoring of *Legionella* in different water systems (Dusserre et al., 2008; National Guidelines for the Control of Legionellosis in Ireland, 2009; Qin et al., 2012; Slimani et al., 2012). Obviously, the presence, even in large concentration, of *Legionella* in cooling towers does not guarantee that the strain responsible for the infection is detected. Supplementary tests such as sequence-base typing must be done to assert the link between clinical isolates and environmental strains (Ginevra et al., 2009). However, this step did not delay the process and had no consequences in the *Legionella* outbreak in Quebec in 2012 and is not criticized in the present paper.

Other laboratories are active in the aerosol science research and also possess the equipment required for *Legionella* aerosol measurement and detection. As previously demonstrated (Blatny et al., 2011), air sampling from various distances from the suspected sources could have helped determining the high concentration zones, circumscribing the area where cooling towers were heavily contaminated and released high concentrations of this pathogen. For example, concentration measurements of the aerosolized *Legionella* over an open air biological treatment plant and along the aerosol plume emitted from this plant demonstrated that decreasing but notable concentrations of *Legionella* could be found hundreds of meters from the plant (Blatny et al., 2011). Models, including wind, temperature and geography data, could have been developed to predict the transport, dispersion and dilution of the airborne contaminants.

The combination of these two approaches could have permitted to determine, within a few days or, at worst, weeks, the epicenter of this outbreak and allow for a much quicker intervention. It is estimated that this type of intervention could have accelerated the source-detection process when it is a known fact that each day gained can be critical in order to halt this type of outbreak.

## Why were the academic laboratories not involved?

Two hypotheses can be proposed as an answer:

1. The research laboratories might not be sufficiently present and known by the governmental organizations;
2. The governmental administration has the tendency to use its own resources when faced with such situation.

The answer, at least for the Quebec City outbreak, is mitigated and each of these two hypotheses has its own merit. On one side, the governmental administration could have made more efforts to build a complete database of skilled scientists in this field (water research, infectious disease specialists, environmental microbiologists, bioaerosols scientists) and use this expertise even if outside of the government administration regular network. As far as water samples, few limitations exist and large bulk samples were available. Distribution of water samples through other provincial government Public Health labs to research institutions could also be a potential way to seek help and collaboration. The lack of a readily available expert database may have impaired this process. Most likely, the governmental administration was not aware of the research capacity and expertise available. Research laboratories have their own responsibility in being not sufficiently known outside their research network. Standard research activities (conferences, workshops) rarely make their way through to the general public and governmental agencies. Research laboratories should make significant efforts toward knowledge transfer and public communication. Since the medical world, which is in charge of public health, and the world of fundamental and applied research are directed by people trained in different academic contexts, they may not all be readily disposed to spontaneous collaboration.

What could be done to prevent such situation from happening again? A database, identifying the numerous governmental para-governmental, private and public organizations possessing an expertise in the field of *Legionella* and, to an extent, in all other agents susceptible of creating situations such as the one experienced in Quebec City in 2012, should be built. The quick integration of a multi-disciplinary special team with diverse field of expertises would have certainly speed up the process and, maybe, even saved a few lives.

This sad story reinforced the importance of the de-compartmentalization of the research laboratories and, unfortunately, the public health office new action plans do not mention this type of integration. If they want to be consulted during such crises, maybe the research experts should build their own database and make it readily available to the numerous governmental agencies. It should be noted that this crisis led to new regulations amending the safety code incorporating provisions relating to the maintenance of cooling towers' water and that use of qPCR will soon be authorized to quantify *Legionella* in cooling towers.

### Conflict of interest statement

The authors declare that the research was conducted in the absence of any commercial or financial relationships that could be construed as a potential conflict of interest.
